# Delineation of B-cell Epitopes of *Salmonella* enterica serovar Typhi Hemolysin E: Potential antibody therapeutic target

**DOI:** 10.1038/s41598-017-01987-8

**Published:** 2017-05-19

**Authors:** Chai Fung Chin, Jing Yi Lai, Yee Siew Choong, Amy Amilda Anthony, Asma Ismail, Theam Soon Lim

**Affiliations:** 1 Institute for Research in Molecular Medicine, Universiti Sains Malaysia, 11800 Penang, Malaysia; 2 Institute for Research in Molecular Medicine, Health Campus, Universiti Sains Malaysia, 16150 Kubang Kerian, Kelantan Malaysia; 3 Analytical Biochemistry Research Centre, Universiti Sains Malaysia, 11800 Penang, Malaysia

## Abstract

Hemolysin E (HlyE) is an immunogenic novel pore-forming toxin involved in the pathogenesis of typhoid fever. Thus, mapping of B-cell epitopes of *Salmonella enterica* serovar Typhi (*S*. Typhi) is critical to identify key immunogenic regions of HlyE. A random 20-mer peptide library was used for biopanning with enriched anti-HlyE polyclonal antibodies from typhoid patient sera. Bioinformatic tools were used to refine, analyze and map the enriched peptide sequences against the protein to identify the epitopes. The analysis identified both linear and conformational epitopes on the HlyE protein. The predicted linear GAAAGIVAG and conformational epitope PYSQESVLSADSQNQK were further validated against the pooled sera. The identified epitopes were then used to isolate epitope specific monoclonal antibodies by antibody phage display. Monoclonal scFv antibodies were enriched for both linear and conformational epitopes. Molecular docking was performed to elucidate the antigen-antibody interaction of the monoclonal antibodies against the epitopes on the HlyE monomer and oligomer structure. An in-depth view of the mechanistic and positional characteristics of the antibodies and epitope for HlyE was successfully accomplished by a combination of phage display and bioinformatic analysis. The predicted function and structure of the antibodies highlights the possibility of utilizing the antibodies as neutralizing agents for typhoid fever.

## Introduction

Hemolysin E (HlyE) is an immunogenic novel pore-forming toxin with hemolytic and cytotoxic activities which is also known as cytolysin A (ClyA) and silent hemolysin A (SheA). Serological data shows that substantial amounts of HlyE are produced by *S*. Typhi and Paratyphi A but not for non typhoidal strains of *Salmonella* such as serovar Paratyphi B and Paratyphi C during human infection^1^. Recent serodiagnostic studies conducted with HlyE under denatured and native conditions showed both forms of HlyE to be antigenic and specific to detect typhoid fever (TF)^2, 3^. Therefore there is a strong indication that HlyE could pose as an important virulence determinant for *Salmonella* Typhi and Paratyphi A pathogenesis.

To date, there is no crystal structure available for *Salmonella enterica* serovar Typhi (*S*. Typhi) HlyE protein in the protein database. Nevertheless, protein sequence analysis reveals that both *E. coli* HlyE and *S*. Typhi HlyE share high similarities at amino acid sequence level. This makes it difficult to isolate antibodies that are specific to *S*. Typhi HlyE. As a basis of comparison, the crystal structure of *E. coli* HlyE can be used for homology modelling. *E. coli* HlyE demonstrates a long rod shape which is a new architecture design for toxin family structures. Based on electron microscopy observation, HlyE molecule is predicted to oligomerize to form a channel that projects out from the membrane, subsequently allowing the formation of a pore^4^. Although *S*. Typhi HlyE is an immunogenic antigen with the potential for diagnostic applications, there is still a gap in knowledge with regards to the antigenic epitopes of *S*. Typhi HlyE that allows the human antibody response to distinguish it from other bacterial HlyE. The ability of typhoid sera to exhibit specificity against *S*. Typhi HlyE suggest the presence of *S*. Typhi HlyE specific epitopes that is identified by typhoid patient sera^3^. Therefore, epitope mapping of the patient sera will provide important insight to these epitopes and the inherent response by the immune system against *S*. Typhi HlyE.

Epitope mapping is a robust approach to elucidate the interaction between antigens and the immune response^5, 6^. This information is vital to identify key antigenic targets/regions that can elicit protective immunity against infections. Therefore the ability to map B-cell epitopes of a specific target antigen is crucial to aid the identification of antigenic regions that are capable of triggering an immune response in the host. The complexity associated with B-cell epitope identification is compounded by the wide spectrum of antigens identified by B-cells. B-cells are capable of recognizing native proteins, glycolipids and polysaccharide antigens based on either linear or structure derived three-dimensional (3D) conformational epitopes^7^. This makes identification of B-cell epitopes for a particular antigen very challenging. The inherent ability of the immune system to generate multiple antibodies against a single antigen compounds greater complexity to the issue. This makes identification of epitopes from a polyclonal pool of antibodies complex.

There are many different approaches used for epitope mapping, however, phage display technology which allows the display of random peptides on the surface of a phage particle is a popular method. This is due to its convenience of use and the ability to identify both linear and conformational epitopes simultaneously using purified antibodies or polyclonal serum^8^. The approach has been effectively used to map epitopes for infectious disease markers in humans^9^. The process would yield a tremendous amount of sequence related data that needs to be processed and analysed against a protein model for a better understanding of structure-function relationship.

The advancement of phage display for epitope mapping is supplemented with the progress made in the field of computational bioinformatics. To analyse a diverse collection of sequences, bioinformatic tools such as Pepitope and EpiSearch web servers are used to map the peptides on 3D antigenic protein structures to clarify specific epitope regions^10, 11^. Such an approach was applied successfully to identify the epitope sequences against RNAIII-activating protein (RAP) of *Staphylococcus aureus* through biopanning with polyclonal anti-TRAP antibodies using a random peptide library^12^. Although challenging, mapping polyclonal antibodies (PcAb) comes with additional information as PcAb recognize multiple epitopes in comparison to monoclonal antibody (mAb) epitope mapping^13^. The developments in structural bioinformatics allows for the elucidation of structure-function relationships of proteins with no available structural information^14^.

A systematic process was designed to take advantage of the existing antibody response from pooled acute human typhoid (Pty) sera that show high specificity towards *S*. Typhi HlyE to delineate the B-cell epitopes of *S*. Typhi HlyE. A random phage display 20-mer peptide library was generated for this purpose. Enrichment of HlyE specific antibodies from Pty sera was done using immunopurification. Biopanning of the phage display 20-mer random peptide library with the enriched PcAb pool was carried out and the enriched peptide sequences were filtered to remove target unrelated peptides (TUPs) before subsequent analysis using several bioinformatic tools. The predicted linear and conformational epitopes were confirmed for reactivity with Pty sera before recombinant mAbs were generated against the HlyE epitopes from a naïve human scFv phage display library. Molecular docking was performed using the 3D model of the *S*. Typhi HlyE to determine the interaction between the generated antibodies with the identified epitopes. Thus, epitope mapping is essential to provide an insight into the interaction between the epitopes with the antibodies and how these interactions could potentially interfere with the folding and function of HlyE. In addition, the approach allows us to reverse engineer the immune response to generate *S*. Typhi HlyE epitope specific mAbs that targets similar epitopes as the natural antibody response exhibited by typhoid patients. The predicted interactions of these antibodies may provide a possible mechanism for the development of neutralizing antibodies for TF.

## Results

### Cloning and expression of rHlyE antigen

The rHlyE was expressed with a predicted molecular mass of approximately 34 kDa and purified with Ni-NTA as shown in Figure S1 (see Supplementary). Immunoblotting was performed to evaluate the antigenicity of the rHlyE against Pty sera with pooled human healty (Phty) as control. To ensure that the fusion tags like His-tag and AviTag was also expressed by the rHlyE, development via immunoblotting using His-tag specific antibodies and streptavidin was carried out as shown in Figure S2 (see Supplementary). Distinct bands were visible for both His-tag, Avi-tag and Pty sera by the rHlyE (approximately 34 kDa) but none for Phty sera.

### Isolation of anti-rHlyE PcAb

The streptavidin bead coupled with biotinylated-rHlyE was evaluated by SDS polyacrylamide gel (see Supplementary, Figure S3). The result shows rHlyE antigen was successfully coupled to the streptavidin bead with a band at approximately 34 kDa for rHlyE. However, a second band at approximately 14 kDa in size is likely to be streptavidin monomer from the bead as shown in lane 4 of the gel. The coupled beads were used to enrich pooled immunoglobulins (Igs) from the Pty sera. The captured Igs were eluted and evaluated using ELISA with rHlyE coated microtiter plate. The immunoassay showed the presence of rHlyE specific antibodies with an optical density readout of 0.75 (see Supplementary, Figure S4). This indicated the successful enrichment of rHlyE specific antibodies from the sera.

### Development and biopanning of the 20-mer random peptide library against rHlyE binding PcAb

**Figure 1:**
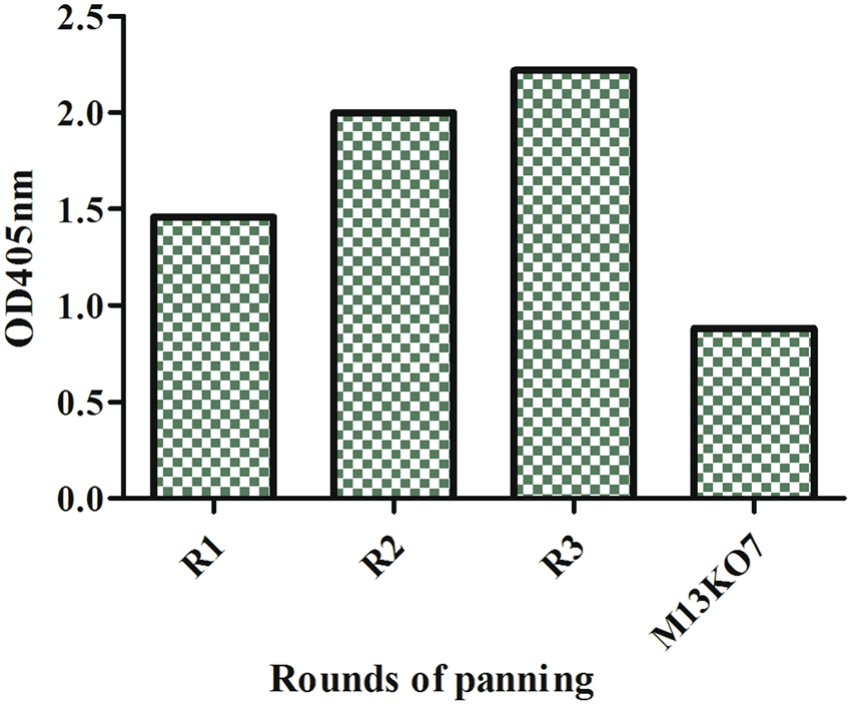
Result of biopanning against anti-rHlyE PcAb. ELISA readouts from round 1–3 were 1.47, 2.00 and 2.22. The experiment was performed with duplicates.

The 20-mer random peptide (NNK) library was constructed with a library size of 3 × 10^9^ CFU/mL. The library was employed for biopanning against the enriched PcAb pool. Three rounds of biopanning was performed and enrichment of clones was evaluated through phage titer (see Supplementary, Table S1) and phage polyclonal ELISA (Fig. 1). The titer of rescued phage from round 1 to 3 indicated approximately 8 folds enrichment of phage. The enrichment of biopanning was further evaluated using polyclonal phage ELISA and the result showed an increment of OD_405nm_ readout from round 1 to round 3. The M13K07 helper phage control showed low levels of non-specific binding in the immunoassay. However, after taking into account the M13KO7 reading as background, the enrichment trend from the first to third round still showed a gradual increase. The results from the phage titer and ELISA showed there was an enrichment of target clones from biopanning.

### Sequence analysis and epitope mapping

After three rounds of biopanning, 60 clones were randomly picked and sequenced. A total of 47 peptide sequences were found to be in-frame with the insert. These peptides were sieved for TUPs using SAROTUP suite. SAROTUP analysis revealed 18 TUPs from the 47 sequences with majority being plastic binders (data not shown). After excluding the TUPs, the remaining 29 peptides were aligned but no direct alignment pattern was visible to match with the HlyE amino acid sequence (data not shown). Subsequently, the 29 peptides were used as input for Pepitope and EpiSearch to locate conformational epitopes on the *S*. Typhi HlyE protein monomer. To ease the data analysis, partitions of overlapped 12-mer peptides were constructed as input for Pepitope web server as PepSurf algorithm can only analyse peptide sequences shorter than 15-mer. An additional epitope prediction web tool, i.e. CBTOPE which is based on the HlyE sequence to predict target conformational epitopes was also applied for analysis. Thus, with the combination of CBTOPE and EpiSearch algorithm, the conformational epitope of HlyE was predicted by considering all the overlapped epitope residues as shown in Figure S5 (see Supplementary), i.e. Y54, S55, Q56, E57, S59, V60, L61, S145, A150, D152, S153, Q154, N212, Q219. This epitope sequence was further supported by PepSurf algorithm which overlaps certain residues of the predicted epitopes, i.e. Y54, S55, Q56, E57, S59, V60, L61. This sequence was designed with an additional amino acid at both the N- and C-terminal ends of the target epitope sequence. Proline and lysine was added to provide a more flexible structure for the conformational epitope, resulting in a final conformational epitope sequence being synthesized as PYSQESVLSADSQNQK. In addition, a linear epitope of HlyE was also predicted based on the overlapped stretch of residues from ElliPro(L) and BcPreds algorithm from BCPREDS as shown in Figure S6 (see Supplementary). Nonetheless, the position of the linear epitope on the surface of HlyE monomer is of utmost importance to increase the accuracy of the prediction. The linear epitope (G180, A181, A182, A183, G184, I185, V186, A187, G188) predicted was found to be situated at the β-tongue of the monomer. The amino acids R172, I173, R174, K175, E176, A177, Y178 and A179 were considered as ‘outliers’ of the linear epitope as they are part of the helix D of the monomer although A177, Y178 and A179 partially overlaps with the starting region of the β-tongue. The overlapped epitope residues that are less than 5 amino acids were ignored as it does not meet the typical minimal length for general linear epitopes. Thus, GAAAGIVAG was predicted to be the most probable linear epitope of HlyE and was synthesized for downstream analysis and application.

### Validation of epitopes by binding immunoassay

**Figure 2:**
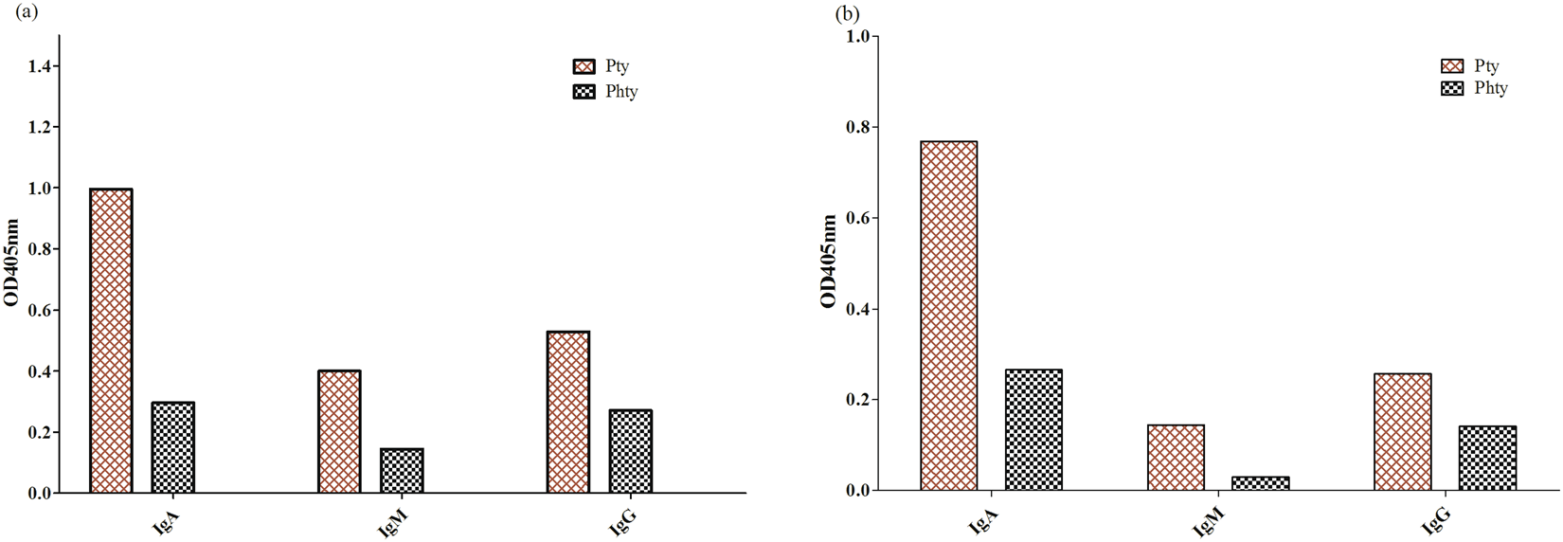
Validation of of rHlyE epitopes. Immunoassay of (**a**) linear rHlyE epitope and (**b**) conformational rHlyE epitope against Pty sera with Phty sera as negative control. The experiment was performed with duplicates.

After a series of analysis, the potential linear and conformational epitopes were identified and synthesized for subsequent validation. The two synthesized peptides, GAAAGIVAG and PYSQESVLSADSQNQK were subjected to an immunoassay against Pty sera with Phty sera as negative control. Results indicated both linear and conformational epitopes were reactive against IgA, IgG and IgM in Pty sera with OD_405nm_ readouts comparatively higher than Phty sera. IgA detection of Pty sera gave readouts at OD_405_ = 1.00 and 0.77 for linear and conformational epitopes respectively with the negative control at OD_405_ = 0.30 and 0.27 respectively. IgG detection for both linear and conformational epitopes (OD_405_ = 0.53 and 0.27) were comparable to IgM detection against typhoid sera (OD_405_ = 0.40 and 0.14) (Fig. 2). The linear and conformational epitopes was also aligned with the sequence of HlyE of *S*. Paratyphi A (UniProt Id:Q93RR6) and *E. coli* (PDB Id: 1QOY:A) to observe for sequence similarity between epitopes obtained with other closely related species (see Supplementary, Figure S7). The regions where the predicted epitopes are located shows sequence variations between the species even when the overall similarities of the HlyE sequence between the species are high. This strengthens the likelihood of the epitopes to be species specific.

### Generation of HlyE epitopes specific mAbs

**Figure 3:**
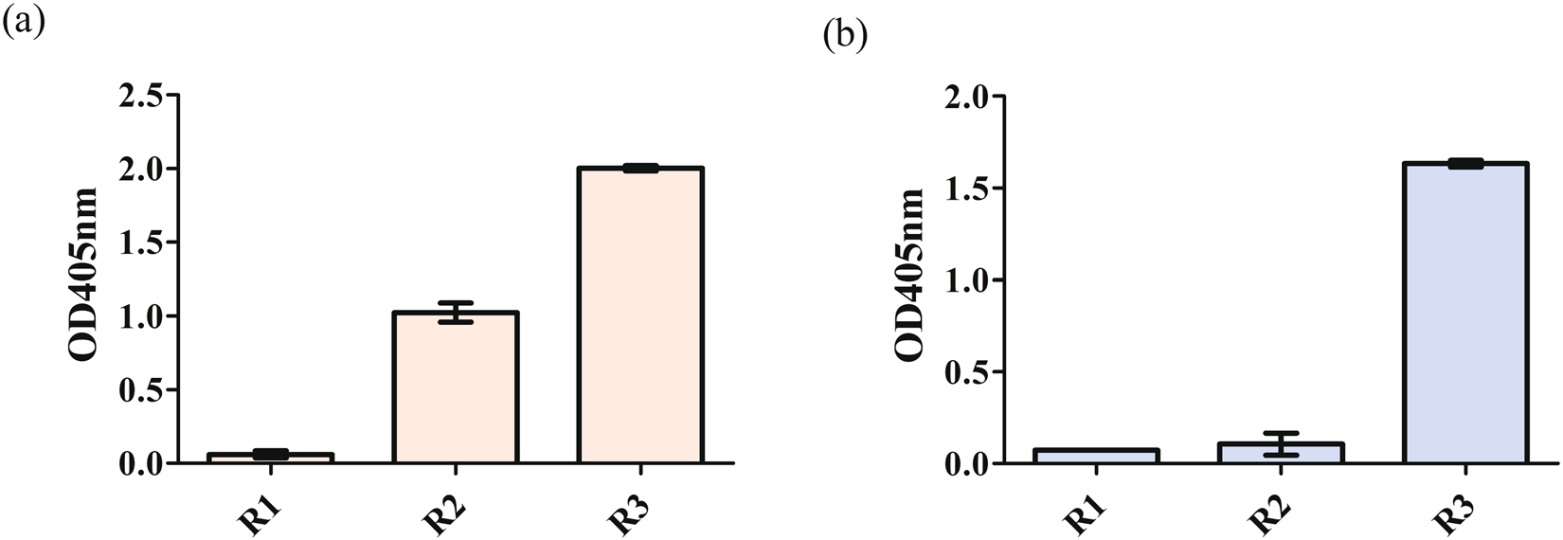
Results of scFv biopanning against the target epitopes. Polyclonal ELISA of biopanning against (**a**) linear epitope and (**b**) conformational epitope of HlyE using naïve scFv library with standard deviation of triplicates shown by error bars.

After both of the linear and conformational epitopes were validated to be antigenic against Pty sera, mAbs against both epitopes were generated using a naïve scFv library. MSIA™ streptavidin D.A.R.T’s® antibody phage library biopanning was performed and phage enrichment was recorded for both targets (Fig. 3). Figure 3a demonstrates the polyclonal ELISA result of the linear epitope for the first to third round of biopanning with OD_405nm_ readouts at 0.06, 1.02 and 2.00 after normalizing with the background. Figure 3b shows the same enrichment pattern with OD_405nm_ at 0.09, 0.24 and 0.88 for the biopanning against the conformational epitope. The results of both polyclonal ELISAs were further supported by phage enrichment titers as shown in Table S2 (see Supplementary). There was an approximate 72 and 92 folds of enrichment from the first to third round of biopanning against the potential linear and conformational epitopes respectively.

After polyclonal enrichment of the specific antibody phage binders against the target epitopes was achieved, 94 monoclonal scFvs were randomly picked and propagated from the third round of biopanning for both linear and conformational epitopes respectively. Monoclonal phage ELISA was performed using selected mAbs against the linear epitope as illustrated in Figure S8(a) (see Supplementary). Nevertheless, for conformational epitope mAbs selection, the template used was rHlyE instead of conformational epitope peptides (see Supplementary, Figure S8b) as we believe conformational epitopes can be recognized more efficiently due to improved folding patterns in the monomeric form. The isolated anti-rHlyE conformational epitope scFvs were further confirmed for their binding by a secondary immunoassay using the synthesized conformational epitope of HlyE. All the possible mAbs with the OD_405nm_ exceeding 0.2 after normalizing with background were further verified by sequencing.

### Validation binding of mAbs against HlyE linear and conformational epitope

**Figure 4:**
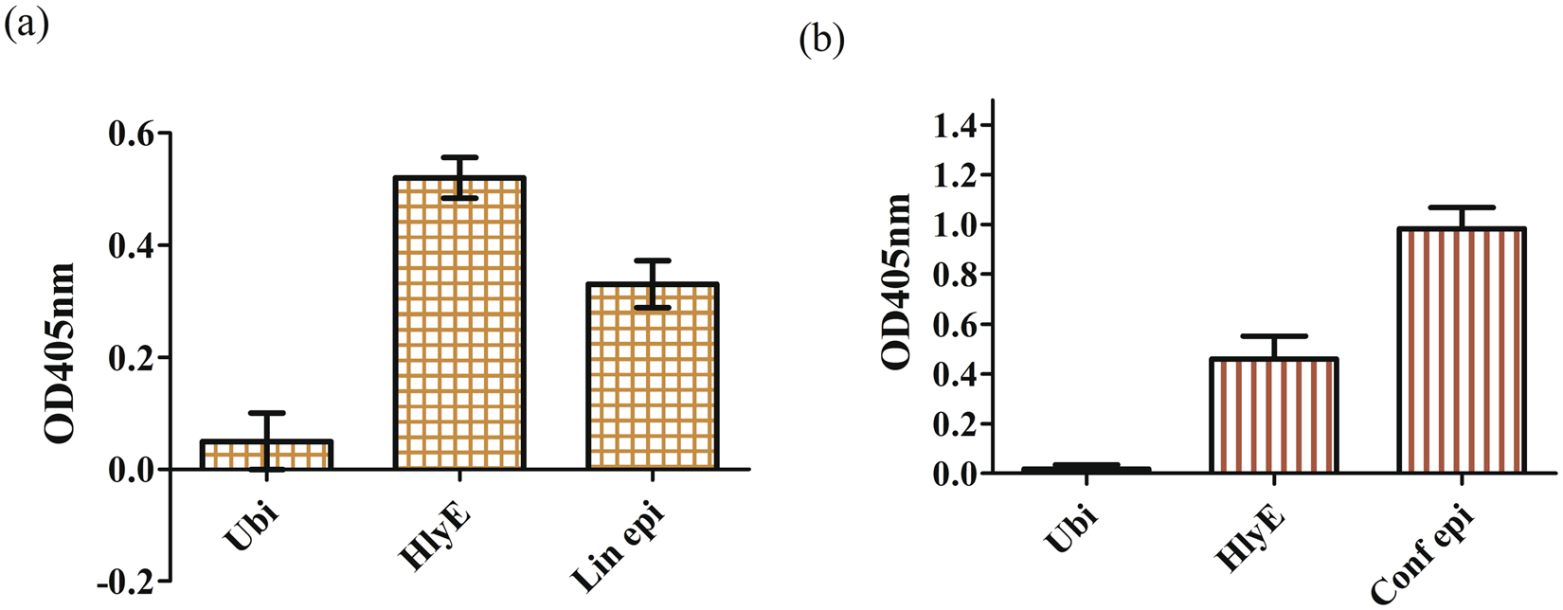
Soluble ELISA of mAbs against HlyE epitopes. (**a**) mAb_L_A11 and (**b**) mAb_C_C2 against ubiquitin, rHlyE and respective target epitopes with standard deviation shown in triplicate.

Sequence analysis identified clones mAb_L_A11 and mAb_C_C2 to be specific against the linear and conformational epitopes of HlyE respectively. The CDR sequences of mAb_L_A11 and mAb_C_C2 are shown in Table S3 (see Supplementary). Furthermore, reactivity of the mAbs against the respective targets and rHlyE was supported by phage and soluble protein ELISA as shown in Figure S9 (see Supplementary) and Fig. 4. Figure S9 describes the binding between phage mAbs towards rHlyE protein and epitopes of HlyE. MAb_L_A11 showed OD_405nm_ readouts of 1.65 and 0.42 against rHlyE protein and linear epitope peptide respectively (see Supplementary, Figure S9a) whereas mAb_C_C2 gave OD_405nm_ readouts of 1.83 and 0.52 against rHLyE protein and conformational epitope peptide respectively (see Supplementary, Figure S9b) with low background. Binding of these mAbs were further confirmed towards the targets in soluble form. This is illustrated in Fig. 4, whereby soluble antibodies are bound to rHlyE (OD_405nm_ = 0.52 and 0.46 for mAb_L_A11 and mAb_C_C2 respectively), target epitopes (OD_405nm_ = 0.33 and 0.98 for mAb_L_A11 and mAb_C_C2 respectively) and ubiquitin (OD_405nm_ = 0.05 and 0.02 for mAb_L_A11 and mAb_C_C2 respectively) as a negative or non-specific binding control after normalizing with background. The expression and purification of mAb_L_A11 and mAb_C_C2 was evaluated by SDS gel electrophoresis for a distinct band at approximately 35 kDa (see Supplementary, Figure S10). The result indicates that mAb_L_All was successfully expressed and purified but mAb_C_C2 was successfully expressed but the purification resulted in low yield of purified product. Therefore, the crude fraction of mAb_C_C2 was used for soluble ELISA. In addition, Western blot and pull down assay were also performed to validate these mAbs (see Supplementary, Figure S11). This was to confirm the binding specificity of the soluble mAbs against HlyE even for clone mAb_C_C2 with the crude fraction. The pulldown assay confirmed mAb_C_C2 to be specific to pull down HlyE as well as the synthesized conformational epitope. In addition, the binding efficacy of mAb_L_A11 and mAb_C_C2 against rHlyE was compared through phage ELISA using a fixed amount of antibody phage particles with a decreasing amount of target antigen as shown in Figure S12 (see Supplementary). The results demonstrated that both phage mAbs were able to bind to rHlyE at 3.125 µg. However, a comparison of the readout indicates that clone mAb_L_A11 provides better binding with a higher readout.

### Identification of binding between mAb_L_A11 and mAb_C_C2 against HlyE target epitopes

**Figure 5:**
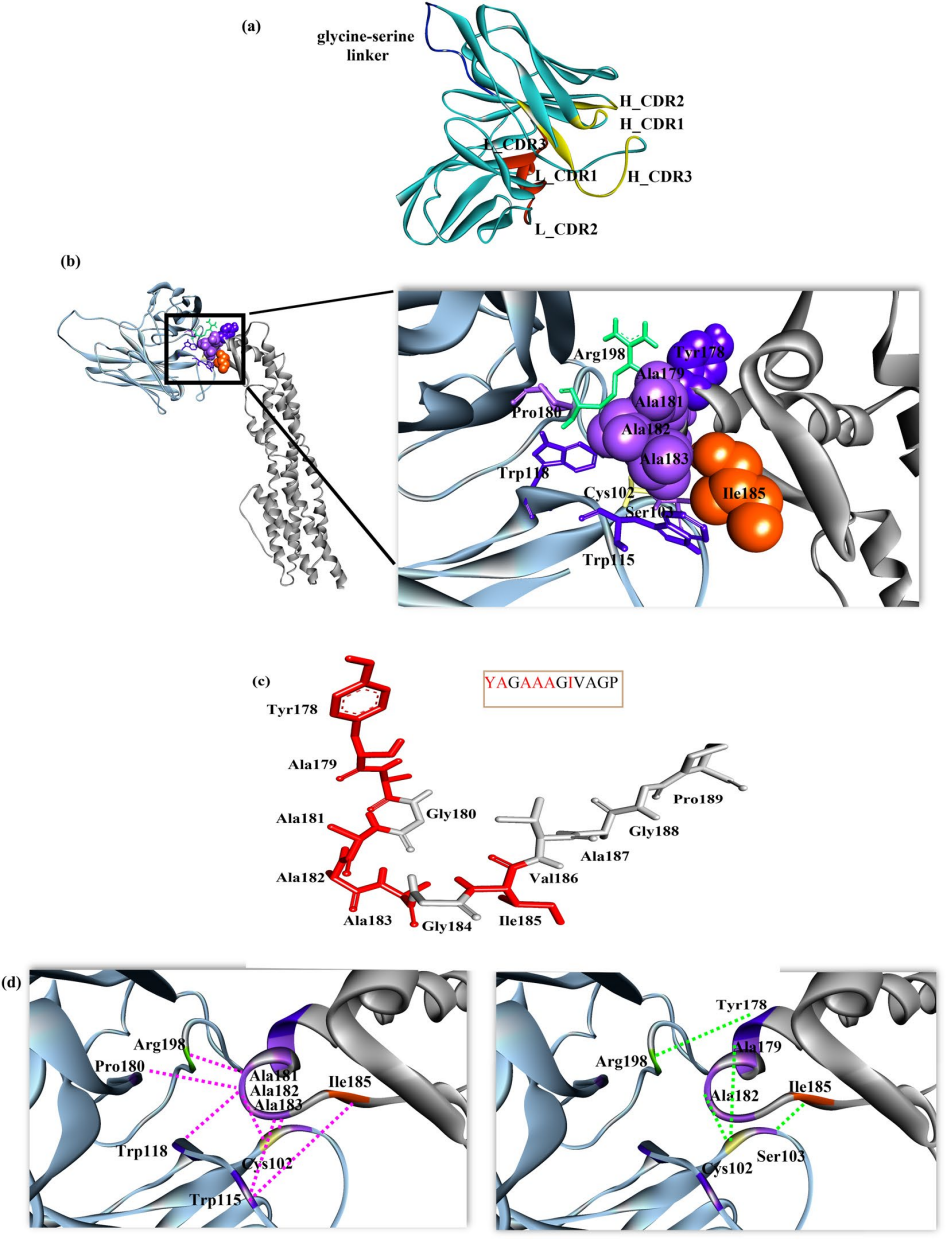
Structural analysis of HlyE linear epitope. (**a**) 3D model of mAb_L_A11 scFv; (**b**) docking pose of mAb_L_A11 (blue) with linear epitope of HlyE (grey) and the insight view of mAb_L_A11 (blue) binding to linear epitope of HlyE (grey). The contacting residues are in stick (paratope) and CPK (epitope) forms; (**c**) contacting residues for HlyE linear epitope with paratopes of mAb_L_A11 are highlighted in red and shown in stick format; (**d**) interaction between mAb_L_A11 (blue) with linear epitope of HlyE (grey) shown with pink and green dotted lines representing hydrophobic interaction and hydrogen bonds respectively.

**Figure 6:**
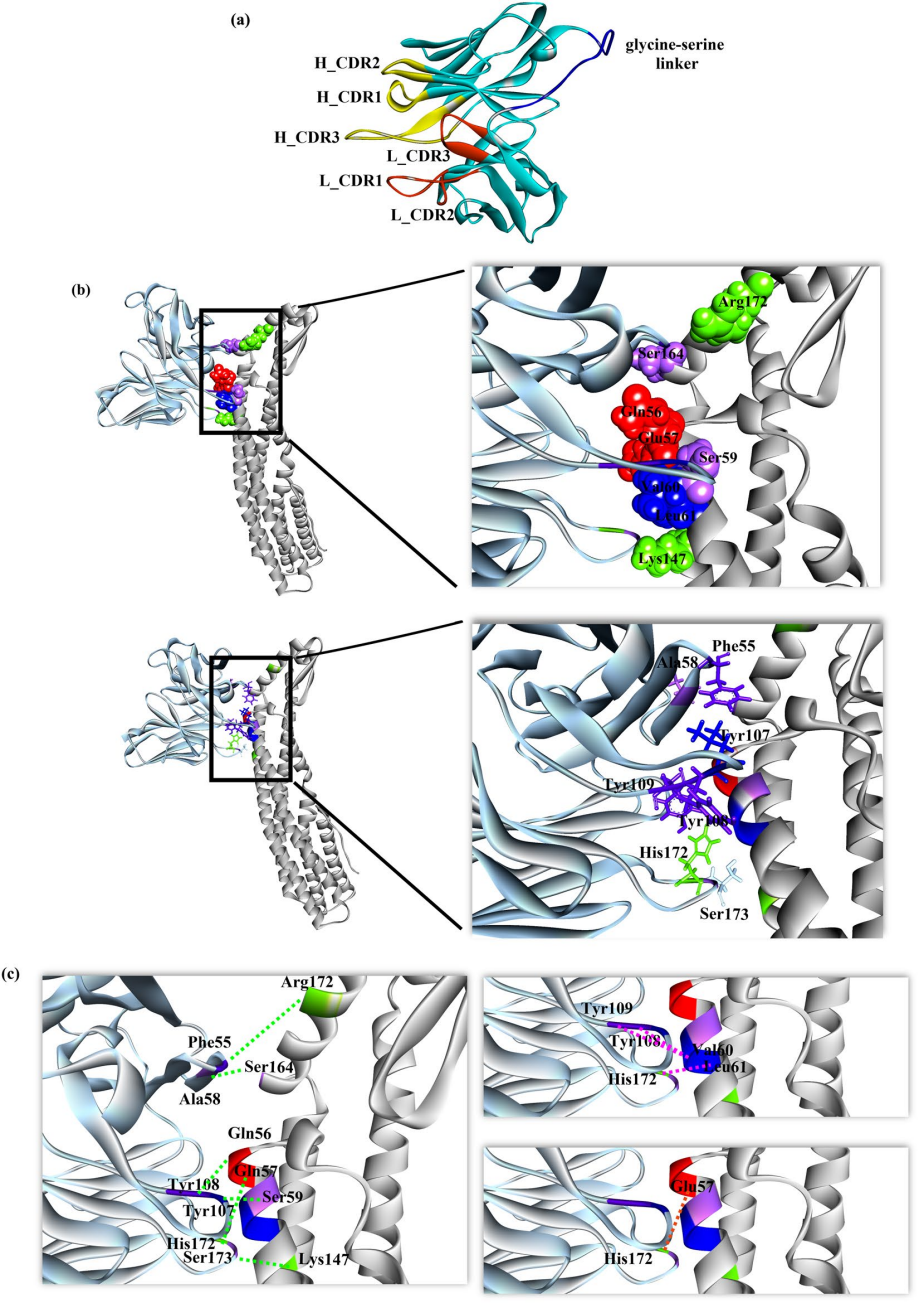
Structural analysis of HlyE conformational epitope. (**a**) 3D model of mAb_C_C2 scFv; (**b**) docking pose of mAb_C_C2 (blue) with conformational epitope of HlyE (grey) and the insight view of mAb_C_C2 (blue) binding to conformational epitope of HlyE (grey). The contacting residues are in stick (paratope) and CPK (epitope) forms; (**c**) interaction between mAb_C_C2 (blue) with conformational epitope of HlyE (grey) with pink, brown and green dotted lines representing hydrophobic bonds, electrostatic interaction and hydrogen bonds respectively.

**Figure 7:**
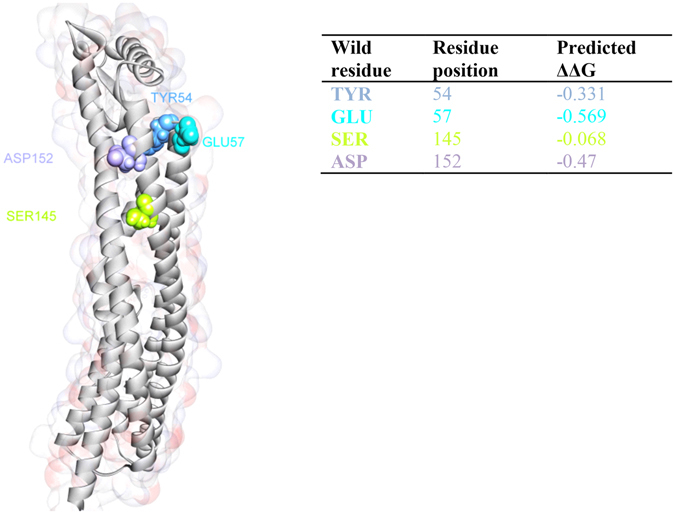
Site-directed mutagenesis analysis of conformational epitope highlighting the most influential residues.

After confirmation of the binding between the mAbs against the target epitopes, homology modelling was performed for mAb_L_A11 and mAb_C_C2 (Figs 5a and 6a) using MODELLER to provide an insight to the binding characteristics between the mAbs and their respective target epitopes using ZDOCK. HlyE was also modelled using MODELLER which was used as input for bioinformatic epitope analysis and as a receptor in molecular docking. The 3D models were chosen based on the lowest DOPE score and percentage of amino acid residues situated in the most favourable regions on the Ramachandran plot. There were no amino acid residues situated on the disallowed regions in the Ramachandran plot. After the 3D monomer structures of both receptor (HlyE) and ligands (mAb_L_A11 and mAb_C_C2) were prepared, molecular docking using ZDOCK was performed. An optimization was also done between epitope sites with CDRs of mAbs to provide a better docking result. Figures 5b and 6b illustrates the docking pose of mAbs against the respective epitopes according to ZDOCK scoring. The view of the respective epitopes and paratopes are shown in CPK and stick format respectively with labels. Figure 5c describes the contacting residues in HlyE linear epitope by mAb_L_A11 scFv. The interaction between mAb_L_A11 and the linear epitope are based on hydrophobic interactions with hydrogen bonds dominating the binding as illustrated in Fig. 5d. From the docking pose, the contacting residues of linear epitope are Y178, A179, A181, A182, A183 and I185 with residues from H_CDR3, L_CDR1 and L_CDR2. On the other hand, Fig. 6c illustrates the interaction between mAb_C_C2 and conformational epitope. The interaction is mainly guided by hydrophobic interactions, hydrogen and electrostatic bonds. The contacting residues of the conformational epitope are Q56, E57, S59, V60, L61, K147, S164 and R172 with residues from H_CDR2, H_CDR3, and L_CDR1. The additional contacting residues identified from the model apart from the predicted epitopes could aid in improving the antibody-antigen interaction. In addition, mutational analysis was performed for each residue (except alanine) of the conformational epitope to determine the role of each amino acid to the binding characteristics of the epitope. Alanine substitution analysis indicated that there are 4 potential residues, which are Y54, E57, S145 and D152 that could be critical epitope residues. This is evident with the reduced calculated binding affinity post-alanine substitution of the four residues (Fig. 7). Analysis shows alanine substitution in other positions yield improved affinities (Table S4, See supplementary). Therefore positions 54, 57, 145 and 152 are critical residues for the conformational epitope.

## Discussion

To our knowledge, there is no report on the antigenic regions or epitopes of the HlyE to date but previous reports suggested there are both linear and conformational epitopes present in *S*. Typhi HlyE^3^. Therefore, identification of the specific linear and conformational epitopes for HlyE is critical to better understand the structure-function relationship. This is important to predict potential strategies for neutralizing the HlyE toxin. The expressed rHlyE was able to react with Pty sera but not with Phty sera which suggest that the rHlyE produced was not structurally compromised and maintains its natural antigenicity.

As we used pooled sera for the experiment, the cocktail of antibodies available in the pooled sera could potentially yield inconclusive results if HlyE specific antibodies were not first isolated. Successful coupling of biotinylated rHlyE to streptavidin beads enable immuno-pulldown of anti-rHlyE PcAb from the Pty sera. As the binding affinity of biotin and streptavidin is resistant to diverse extreme elution conditions, isolation of the HlyE specific antibody population was achieved by elution under low pH conditions and with immediate neutralization. The ability to isolate HlyE specific antibodies from the pooled sera was confirmed with strong immunoassay readouts.

The introduction of a 20-mer random peptide library was to ensure better coverage of conformational epitopes which usually falls in the between the range of 10 to 22 residues^5^. The system was designed with the use of a phagemid instead of the commercially available phage systems which provides shorter peptide sequences. This brings together the advantages of the selectivity of the phagemid system and also the convenience of biopanning for protein interaction analysis with longer peptides. The biopanning experiment recorded increments of around 8 folds in between the biopanning rounds which is in agreement with the phage ELISA data that suggests the presence of enriched peptide binders against anti-rHlyE PcAb. The enrichment of only 8 folds in three biopanning rounds is possibly due to the complexity of the PcAb pool as opposed to conventional mAb biopanning for mapping experiments. Large diverse peptide binders with lower folds of enrichment will be achieved towards the binding of PcAb pools instead of a mAb.

Subsequently, a total of 60 clones from the third round of biopanning were randomly selected for sequence analysis. Sequencing results of the 60 clones yielded 47 useful sequences for further analysis as the remaining 13 clones were out of frame. This is common during the cloning process of the peptide library as the randomized regions could generate unwanted annealing partners resulting in frame-shifted clones. The 47 peptides were then filtered using SAROTUP suite to remove any non-specific or surface binders. A total of 18 TUPs, mainly plastic binders were identified using SAROTUP and were excluded to avoid noise and false positive in subsequent analysis. The presence of amber stop codons in 20-mer pepides is an expected phenomenon as the peptide library used was designed with the NNK degeneracy. All the clones with amber stop codons were substituted with glutamine as TG1 cells (amber suppressor strains) will automatically replace the amber stop codon with glutamine^15^.

Previous work with the HlyE antigen from *S*.Typhi showed that typhoid patient sera was able to react with HlyE under both native and denatured forms^2, 3^. This is a clear indication that both linear and conformational epitopes are present for HlyE. The accuracy of predictions by computational methods has shown acceptable success rates that provides a means to anticipate the possible available epitopes from a specific target antigen. The prediction analysis of *S*. Typhi HlyE using ElliPro, BCPRED and CBTOPE was carried out to predict possible linear and conformational epitope^16–18^. BCPRED employs a novel approach using sub-sequence kernel to predict linear B-cell epitopes from proteins^17, 19^ and predicts a 20-mer peptide, RIRKEAYAGAAAGIVAGPFG with the highest score. Interestingly, ElliPro web tool predicts the same stretch but with a longer amino acid sequence, GKLLALDSQLTNDFSEKSSYFQSQVDRIRKEAYAGAAAGIVAG (see Supplementary, Figure S6). We speculated that a linear epitope could be present in this stretch of amino acid sequence. Therefore by overlapping these two peptides, a shorter sequence, RIRKEAYAGAAAGIVAG was obtained. After looking into the monomer structure of *S*. Typhi HlyE, the RIRKEAYA motif was found to be part of the D helix, whereas GAAAGIVAG is located on the β-tongue region of the toxin which normally functions to bind and target cell lysis by forming pores in the membrane surface^4, 20, 21^. Taking into consideration the linear epitope prediction and the structure of the toxin, a deduction was made whereby the linear epitope of HlyE would encompass G180, A181, A182, A183, G184, I185, V186, A187 and G188 although multiple sequence alignment performed shows no resemblance between 29 in-frame biopanning peptide sequences with the HlyE gene of S. Typhi Ty2 strain.

At the same time, CBTOPE web tool was also employed to generate conformational B-cell epitopes of HlyE based on bioinformatic prediction from its primary amino acid sequence^22^. As predictions that are solely dependent on bioinformatic analysis do not provide sufficient data to support newly found epitopes, a combination of laboratory experiments and computational analysis would be able to help enhance the validity of the data. From previous biopanning and filtration of TUPs, 29 affinity selected peptide sequences were used as input in EpiSearch and Pepitope web servers to analyse potential conformational *S*. Typhi HlyE epitopes. Therefore, we combine CBTOPE and EpiSearch analysis by overlapping these 2 results with an effort to obtain a consensus sequence for conformational epitope from wet and dry lab experimental data. A conformational epitope was deduced as Y54, S55, Q56, E57, S59, V60, L61, S145, A150, D152, S153, Q154, N212, Q219 and this was further supported by PepSurf algorithm from Pepitope web server (see Supplementary, Figure S5). EpiSearch is a widely used web tool to predict conformational epitopes located on the surface of antigens with its highest scoring patches tested in experimental models covering more than 50% epitope residues in antibody-antigen complexes found in X-ray crystallography^11^. Thus, the output generated by EpiSearch would generally provide higher confidence. PepSurf algorithm was proposed to be efficient in searching for highly similar peptides to the target antigen through 3D paths and brings them together via folding. This will result in the most significant alignments being clustered and the locations of the epitopes are subsequently inferred^23^. Although the target used was mainly mAbs, using a PcAb pool as template was also possible with satisfactory results obtained from the PepSurf algorithm for epitope prediction. The synthesized conformational epitope of HlyE for downstream analysis was PYSQESVLSADSQNQK with the additional proline added to the N-terminal of the conformational epitope due to its non-polar characteristic that will not change the overall charge of the peptide but at the same time provide a longer sequence for structural folding. On the contrary, lysine as the basic amino acid was added to the C-terminal of the epitope sequence in order to improve the solubility of the peptide in addition to structural folding. This is because to design a soluble peptide, it is preferable to have 1 out of 5 amino acids to be charged. In addition, lysine is also one of the preferred amino acid in carboxyl end capping for a better α helix stabilization in short peptides^24^. The conformational epitope was deduced to be situated at the B, C and F helices of the HlyE monomer which may encounter specific antibody responses during infection.

Both linear and conformational epitopes, GAAAGIVAG and PYSQESVLSADSQNQK epitopes were evaluated for their antigenicity by immunoassay against Pty sera. The result showed both epitopes were reactive against Pty but not with the Phty control. This indicates both peptides are possible HlyE epitopes of *S*. Typhi with a higher IgA detection as compared to IgG and IgM. Significant IgA detection in anti-rHlyE Pty sera could be due to *S*. Typhi infections that are concentrated at the gastrointestinal mucosa that would induce intestinal mucosa immune responses^25^ for IgA secretion to react with the target antigens. Nonetheless, IgG detections of both epitopes are slightly higher than IgM detection which is in agreement with previous report^3^. IgG detection of anti-HlyE typhoid sera was also verified in Bangladeshi patients especially from acute to convalescent stage^26^. This suggests that the epitopes present could be used for detection at different stages of typhoid infection. Alignment between this *S*. Typhi potential epitopes with *S*. Paratyphi A show both of them share similar epitope sites as both are typhoidal strains. Nonetheless, there are deviations of two amino acids for both linear and conformational epitopes between *S*. Typhi and *E. coli* strains which lead to the possibility that these epitopes may exhibit low cross reactivity towards other species.

After the immunogenicity of both HlyE linear and conformational epitopes against Pty sera were validated, mAbs against both epitopes were generated using the MSIA™ streptavidin D.A.R.T’s® antibody phage library biopanning approach^27^. This biopanning method was previously used to enrich mAbs against HlyE. The similar process was applied here to enrich mAbs against both conformational and linear epitopes of HlyE using an in-house naïve scFv library^28^. All mAbs that exceeded the minimal OD_405nm_ readout were sent for sequencing and two mAbs, mAb_L_A11 and mAb_C_C2 were identified as potential mAbs against the linear and conformational epitopes respectively. Although there were numerous clones that fulfilled the requirement in terms of the OD_405nm_ readout, sequencing results revealed only these two clones showed complete scFv sequences. The binding of these two mAbs were further confirmed against the target HlyE epitopes by repackaging them as phage as well as expressing them as soluble antibodies. The ELISA results reveal mAb_L_A11 and mAb_C_C2 binds better towards rHlyE compared to the synthesized epitope peptides. This could be due to lower binding affinities of peptides towards the ELISA plate surface which could hinder sufficient surface area for antibodies to bind. A larger sized recombinant version of the antigen would provide better binding resulting in an improved readout. However, we observed an exception for soluble mAb_C_C2 scFv that binds better with the conformational epitope peptide as compared to rHlyE. All the binding variation between native rHlyE and epitope peptides may be subjected to immunological cross-reactivity between proteins and homologous peptides depending on a balance of local disorder in the proteins as well as propensity towards unique secondary structure of the epitope peptides situated on the antigenic location^29^. The validation of mAb_L_A11 and mAb_C_C2 were also confirmed by denaturing Western blot and pull-down assays. A comparison between the efficacy of both mAbs in detecting rHlyE was demonstrated using phage ELISA. The results showed mAb_L_A11 was able to bind better towards rHlyE with an amount as low as 3.125 µg when compared to mAb_C_C2. The higher binding efficacy of mAb_L_A11 with the rHlyE antigen suggests that mAb_L_A11 has a higher affinity when compared to mAb_C_C2 scFv.

Mapping of HlyE linear and conformational epitopes were further illustrated by performing molecular docking. Since the binding between mAb_L_A11 and mAb_C_C2 and respective epitopes were validated, 3D models of these antibodies (ligands) and HlyE antigen (receptor) were also modelled by MODELLER and docked to provide an insight to the interactions involved. The molecular view of the docking pose for the linear epitope with mAb_L_A11 complex shows that hydrophobic interaction dominates the interaction over hydrogen bonding. This is probably due to the fact that hydrophobic interactions are mainly involved in the primary interaction between epitope-paratope which could overcome the overall hydrophilic repulsion to bring the complex together^30^. Moreover, this could be partly due to the position of the proposed linear epitope being situated at the beginning of the β-tongue which is hydrophobic in nature. This hydrophobic region was proposed to have the membrane penetration ability that will bind and lyse the target cells through pore forming^4, 20, 21^. This leads to the possibility that mAb_L_A11 could potentially function as a candidate for pathogen neutralization. The interaction of mAb_L_A11 to the β-tongue could potentially function to inhibit the HlyE monomer from penetrating the target membrane, thus, preventing cell lysis. Besides, the docking pose also reveals 2 additional amino acids, K178 and A179 that could be involved in the binding towards mAb_L_A11 although it was not in the predicted linear epitope. This is likely due to their position being situated in the D helix which is at the start of the β-tongue. However, this also indicates that further refinement of the epitope region could be required to obtain a higher accuracy.

In addition, the interaction between the proposed conformational epitope with mAb_C_C2 shows that hydrophobic interaction, electrostatic and hydrogen bonds are all involved. Unlike the linear epitope, the proposed conformational epitope spans through the B, C and F helices. As there are limited literature found in mapping conformational epitopes using phage display technology with linear epitopes being more common, the results also shows the ability of a combinatorial approach involving peptide phage display and bioinformatics to be used to map conformational epitopes for PcAb. According to the docking pose, mAb_C_C2 could exert steric hindrance that may block the rearrangement of the monomer (rearrangement into 3 helix bundles instead of four)^31^ to form an oligomer. This is critical as the oligomeric form of HlyE is responsible for pore formation after cell wall penetration. Therefore, further infections can be contained by restricting pore formation on cells. In addition, each residue of the HlyE conformational epitope was mutated with alanine in order to investigate the importance of the each residue in the antibody recognition. The result suggest that there are 4 potential residues which greatly affect the binding. The critical residues were identified to be Y54, E57, S145 and D152. This further strengthen our finding that a conformational epitope exist in the HlyE antigen which can be valuable for potential diagnostic or therapeutic applications.

In conclusion, the systematic identification of the HlyE epitopes as well as mAb development aided by computational based analysis can provide in-depth information on the possible function of HlyE in typhoid pathogenesis. More importantly, it allows us to replicate the natural antibody responses of infected individuals to generate *S*. Typhi HlyE specific antibodies that identify similar epitopes. In addition to that, the detailed interaction between the generated mAbs with the HlyE model also allows a hypothetical analysis of potential therapeutic strategy for typhoid infections. The mAbs generated in this work could potentially be evaluated for efficacy as neutralizing antibodies in the future.

## Methods

### Expression and validation of rHlyE antigen

Expression of biotinylated rHlyE was performed according to Ong *et al*., 2013^32^. Purified fractions were subjected to sodium dodecyl sulfate-polyacrylamide gel electrophoresis (SDS-PAGE) and Western blot to detect the presence of His-tag, biotinylation and immunogenicity towards Pty sera.

### Isolation/Enrichment of anti-rHlyE PcAb

The Pty sera were obtained from the serum bank collection of Institute for Research in Molecular Medicine (INFORMM Biobank), USM. Magnetic separation of HlyE specific antibodies was carried out by coupling biotinylated-rHlyE to paramagnetic streptavidin beads (Chemicell, Germany) o/n with gentle agitation. Then, 100 µl Pty sera was incubated with 200 µl HlyE bound beads with gentle shaking for 1hr at room temperature (RT). Subsequently, Igs bound to the rHlyE were eluted using acid elution (0.2 M glycine-HCl, pH 2.2) with agitation at 700 rpm for 15 min and immediately neutralized to pH7 with 1 M Tris-HCl. A sandwich based ELISA of the antigen and antibody was performed to test sensitivity and specificity of the eluted Igs according to Crowther and Walker^33^ in which the antibodies will serve as the target for biopanning.

### Generation of 20-mer random peptide library

20-mer random phage display library was constructed according to Adey and coworkers with slight modifications^34^. Random peptide oligonucleotide inserts and a reverse primer were synthesized (1^st^ Base, Malaysia) with the sequence 5′-CCGGCCATGGCC(NNK)_20_GCGGCCGCATAGACTGTT-3′ and 5′-AACAGTCTATGC-3′ respectively. The degenerate oligonucleotides, which consist of 20 repeating NNK codon with N being an equal mixture of A, G, C and T, each K is an equal mixture of G and T. This allows the coding of all available amino acids and one stop codon^35^. Each synthesized oligonucleotide was assembled into double stranded DNA by a filling in reaction using Klenow fragment (NEB). The degenerate sequence was cloned into pLABEL phagemid vector with NcoI and NotI restriction sites and introduced into *E. coli* XL1-Blue competent cells (Agilent Technologies, CA) through electroporation. Approximately 400 transformations were done to construct the random peptide library, and followed by recovery and storage of the library.

### Biopanning of anti-rHlyE PcAb

Target anti-rHlyE PcAb was panned using the 20-mer NNK phagemid library (3 × 10^9^ CFU/mL) constructed earlier to obtain potential enriched epitope sequences. The biopanning protocol was according to Chin *et al*., 2015, with slight modifications^36^. Three successive biopanning rounds were performed with 3 µg anti-rHlyE PcAb pool in bicarbonate buffer (0.1 M NaHCO_3_, pH8.6) coated on costar microtiter plate (Corning, USA) at 4 °C with 700 rpm agitation. After washing three times with phosphate buffered saline (PBS, pH7.4) containing 0.1% (v/v) Tween (PBS-T), anti-rHlyE PcAb coated well was incubated with blocking buffer (3% w/v bovine serum albumin in PBS, 3% BSA) for 1 h at RT with 700 rpm agitation to reduce non-specific binding. The well was washed three times with PBS-T. Subsequently, phage particles (10^12^ CFU/mL) were diluted in PBS buffer and added into the coated plate for 1 h incubation at RT with 700 rpm agitation. The plate was washed three times with PBS-T to remove unbound phage. The bound phages were eluted by 15 min incubation with 100 µl of 0.2 M glycine-HCl, pH2.2 with 700 rpm agitation and immediately neutralized with 1 M Tris HCl, pH 9.1. The eluted phages were used to infect an exponentially growing TG1 (Agilent Technologies, CA) culture (OD_600nm_ of 0.5) and titration of rescued phage was performed for each round of biopanning. Packaging and amplification of the three successive rounds of biopanning were then preformed.

### Phage ELISA for evaluation of relative binding of phage to anti-HlyE PcAb pool

After 3 rounds of selection, the enrichment and specificity of the phage were assessed by ELISA. Briefly, 10^13^ of phage particles from the peptide library and rounds of biopanning were coated on the microtiter plate o/n at RT with agitation at 700 rpm in PBS buffer. M13K07 (NEB, USA) was also coated as negative control and blocking buffer (2 g blocker non-fat dry milk in 100 ml PBS-T, 2% PTM) as background control. After washing three times with PBS-T, the wells were blocked with 2% PTM (1 h, 700 rpm) to reduce non-specific binding. The plate was washed thrice with PBS-T, subsequently the Pty sera and Phty (1:100 in 2% PTM) were incubated for 1 h at RT. Bound IgGs were then detected by horseradish peroxidase-anti-human IgG (Sigma Aldrich) (1:2500 in PTM). Lastly, 2,2′-Azino-bis(3-ethylbenzothiazoline-6-sulfonic acid) diammonium salt (ABTS) (Amresco, USA) developing solution was used to detect bound IgGs carrying the peroxidase enzyme that covert the substrate into a colour product. Absorbance reading at 405 nm (OD_405_) was also taken with a microplate reader (Thermo Scientific Multiskan Spectrum).

### Nucleotide Sequencing

Library phagemid clones were plated out from the third round of biopanning after the enrichment of peptide sequences against anti-HlyE PcAb pool. 60 random clones were randomly picked and sequenced for further analysis.

### HlyE model construction

The sequence of HlyE from *S*. Typhi was obtained from UniProt (id: Q8Z727) and was further searched against Basic Local Alignment Search Tool (BLAST)^37^ for suitable templates in RCSB Protein Data Bank (PDB). Sequence alignment with template (PDB ID: 1QOY) to model the HlyE monomer was performed by Clustal Omega^38, 39^. The 3D model for the protein was performed by MODELLER 9v14^40, 41^ with a total of 100 models being randomly generated. The model with the best DOPE^42^ was evaluated by Ramachandran plot from PROCHECK^43^. This HlyE 3D model generated will serve as input for structural analysis and molecular docking of the identified epitopes.

### Epitope prediction

Prediction of linear antigenic regions of HlyE was carried out using BCPREDS web server (http://ailab.cs.iastate.edu/bcpreds/)^17^ using an epitope length of 20-mer with a cut off value exceeding 0.8. Putative antigenic determinants were also analysed by the ElliPro prediction web tool (http://tools.immuneepitope.org/tools/ElliPro)^18^. Based on the 3D structure of the HlyE protein antigen, linear epitope residues were obtained with default input setting (distance *R* set at 6 Å; score *S* set at 0.5). Prediction of continuous antigenic residues of HlyE protein that could possibly interact with the antibodies of interest was carried out through CBTOPE web server which is solely based on the primary sequence of the antigen (http://www.imtech.res.in/raghava/cbtope/)^16^.

### Epitope mapping analysis from previous phage display peptides

Sequences from the clones obtained from biopanning was filtered using the tools in the SAROTUP suite to exclude any possible TUPs^44^ before proceeding as input for mapping and aligned with the HlyE sequence to identify overlapping sequences. Then, the ‘filtered’ peptides were then mapped to the 3D structure of HlyE monomer from *S*. Typhi using the Pepitope^10^ and EpiSearch web tool^11^ to analyse the most probable conformational epitopes present on the surface with the respective algorithm and default setting. The potential linear and conformational epitopes were analysed further based on overlapping residues from ElliPro, BCPREDS, CBTOPE, Pepitope and EpiSearch bioinformatic tools.

### Verification of HlyE epitopes

Two potential epitopes, GAAAGIVAG and PYSQESVLSADSQNQK were synthesized (1^st^ base, Malaysia) with biotin modification at the N -terminal. These two potential linear and conformational peptides were subjected to an immunoassasy with Pty sera to test its antigenicity with Phty sera as negative control. Briefly, 20 µg of peptides in PBS buffer were coated o/n at 4 °C with blocking buffer as background control. After washing three times with PBS-T, the wells were blocked with 5% BSA (1 h, 700 rpm) to reduce non-specific binding. The plate was washed thrice with PBS-T, subsequently Pty and Phty sera (1:50 in BSA) were incubated for 1 h. Detection of peptides binding to the sera was done with horseradish peroxidase-anti-human IgA, IgG and IgM (1:2500 in BSA) respectively. Lastly, ABTS developing solution was used to detect bound Igs carrying the peroxidase enzyme and absorbance reading at OD_405nm_ was also taken with a microplate reader.

### Biopanning of the generated epitopes using naïve scFv library

The identified antigenic peptides were then subjected to a semi-automated biopanning protocol, i.e., MSIA™ streptavidin D.A.R.T’s® antibody phage library biopanning to generate scFv mAbs against the peptides^27^. Biotinylated linear and conformational peptides at 100 μg in PBS buffer was loaded to the MSIA™ Streptavidin D.A.R.T’s® by continuous aspiration and dispensing instead of carbonate buffer due to the stability as well as solubility of the peptides in PBS buffer. 5% PTM was used as blocking buffer as a higher percentage of blocking agent is needed to block the background from the system. In addition, an increase of aspiration and dispensing cycles in the elution steps (500 instead of 300 cycles) was performed to optimize the elution of bound phages from the MSIA™ D.A.R.T’s® system. Phage titer and polyclonal ELISA were also performed to observe the enrichment pattern of biopanning according to Chin *et al*., 2016^27^ with 5% PTM as a blocking agent.

### Selection and validation of mAbs against generated epitopes

Monoclonal scFv antibody selection, expression and ELISA were done according to Chin *et al*., 2016^27^ after three successive rounds of biopanning. Both rHlyE antigen and peptides were used to test the binding of the selected mAbs in phage and soluble form by ELISA. The potential epitope-specific scFv sequences were also verified by DNA sequencing. In addition, bacterial expressions of mAbs were also performed to obtain soluble forms of the scFv. The validated epitope-specific mAbs were transformed into *E. coli* non-amber suppressors, Top10 F’ and BL21 (DE3). His-tag purified fractions of the mAbs were subjected to SDS-PAGE and validated by Western blot before use in ELISA.

### Phage ELISA to compare binding efficacy of mAbs against rHlyE

A total of 10^10^ phage mAb_L_A11 and mAb_C_C2 particles were coated on the microtiter plate o/n at 4 °C in PBS buffer. After washing three times with PBS-T, the wells were blocked with 3% PTM for 1 h at 700 rpm. The plate was washed thrice with PBS-T and subsequently added with a serial dilution of rHlyE in 3% PTM (0.39–50 µg). Background control well was added with only 3% PTM. The plate was incubated for 1 h at RT, 700 rpm. The plate was washed thrice with PBS-T and bound rHlyE was detected by incubating for 1 h with streptavidin horseradish peroxidase (1:5000 in PTM). Finally, the peroxidase enzyme was detected using ABTS and absorbance readings at 405 nm were taken with a microplate reader.

### Homology modelling of monoclonal A11 and C2 scFv

The verified mAbs that specifically bind to both linear and conformational epitopes were subjected to comparative modelling using MODELLER 9v14^40, 41^ to construct 3D scFv models. Multi-template modelling was performed to model the scFv as the sequence identity between templates and target scFv is below 70%. Templates used for the A11 scFv are PDB ID: 5c6w, 2ykl, 5cgy, 3wlw, 4xak, 4gsd, 4ydk, 4osu, 4jdt, 1yrw, 4k91; C2 scFv with PDB ID: 3fno, 3juy, 4fz8, 4hk0, 4lri, 5c6w, 3gkw, 3gnm, 3ld8, 4lf3. A total of 100 models were randomly generated for each respective model. The model with the best DOPE score^42^ was evaluated by Ramachandran plot from PROCHECK^43^. Loop refinement was performed by MODELLER to optimize the model with residues situated at disallowed region in Ramachandran plot. A11 and C2 3D models were used for subsequent docking analysis.

### Docking of A11 and C2 scFvs with epitopes of *S*. Typhi HlyE

The 3D models of A11 and C2 scFvs were submitted to ZDOCK^45^ module against the linear and conformational regions of HlyE monomer for molecular analysis. Frameworks from both scFvs were blocked to provide a better surface contact between the complementary determining regions (CDRs) and the target epitopes. The 2000 complexes generated from the software or initial docking predictions were re-ranked by ZRANK^46^. The pose with the highest score was selected as the predicted complex and analysed by Discovery Studio Visualization 4.5 (Biovia Dassault System, USA) for molecular interaction analysis and graphical interpretation.

### Prediction of antibody–antigen affinity changes upon mutation

Each residue on the conformational epitope was mutated to alanine for the prediction of antibody-antigen affinity changes using mCSM-AB. mCSM-AB is an online bioinformatic server (http://bleoberis.bioc.cam.ac.uk/mcsm_ab/prediction) that relies on graph-based signatures to predict antibody–antigen affinity changes upon mutation^47^.

## Electronic supplementary material

Supplementary data
