# Supplementary material for: Delineation of B-cell Epitopes of Salmonella enterica serovar Typhi Hemolysin E: Potential antibody therapeutic target
Source: Sci Rep. 2017 May 19;7:2176. doi: 10.1038/s41598-017-01987-8 (PMC5438399; doi:10.1038/s41598-017-01987-8)
Supplement: Supplementary file 1 — Supplementary data [file 41598_2017_1987_MOESM1_ESM.doc]

**Delineation of B-cell Epitopes of *Salmonella* enterica serovar Typhi Hemolysin E: Potential antibody therapeutic target**

**Chai Fung Chin^1^, Jing Yi Lai^1^, Yee Siew Choong^1^, Amy Amilda Anthony^2^, Asma Ismail^2^ and Theam Soon Lim^1,3^,***

^1^Institute for Research in Molecular Medicine, Universiti Sains Malaysia, 11800 Penang, Malaysia

^2^Institute for Research in Molecular Medicine, Health Campus, Universiti Sains Malaysia, 16150 Kubang Kerian, Kelantan, Malaysia

^3^Analytical Biochemistry Research Centre, Universiti Sains Malaysia, 11800 Penang, Malaysia

*To whom correspondence should be addressed:

 Tel: +604-653-4852, Fax: +604-653-4803, Email: [theamsoon@usm.my](mailto:theamsoon@usm.my)

**Supplementary data**


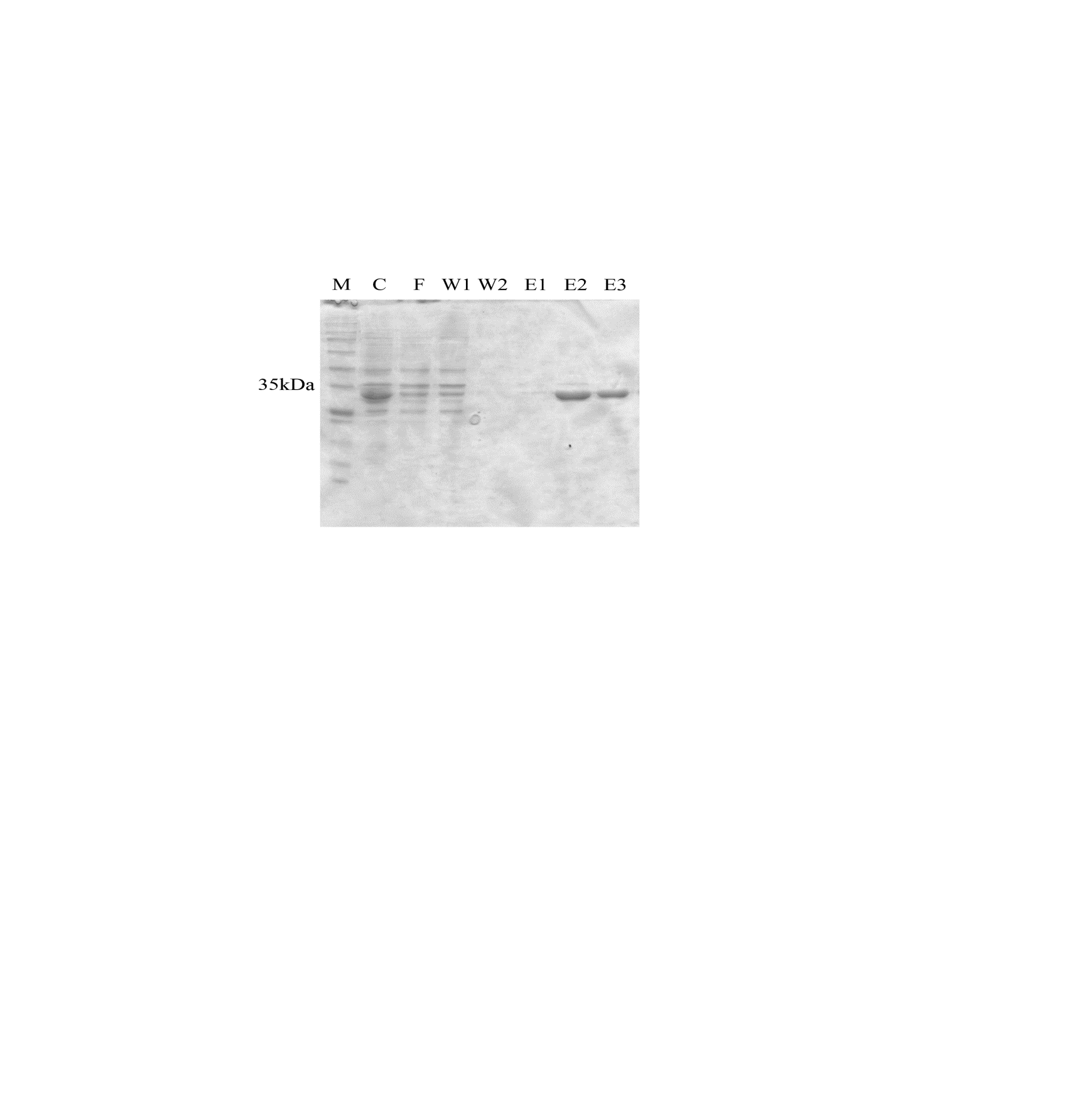


**Figure S1. Coomassie blue staining of the rHlyE** **antigen**. The 12% SDS-polyacrylamide gel showing HlyE antigen with M indicates protein marker; C denotes crude of lysate protein; F denotes flow-through of the lysate protein; W1 denotes first wash; W2 denotes last wash, E1-3 denotes lysates after passage through affinity column.


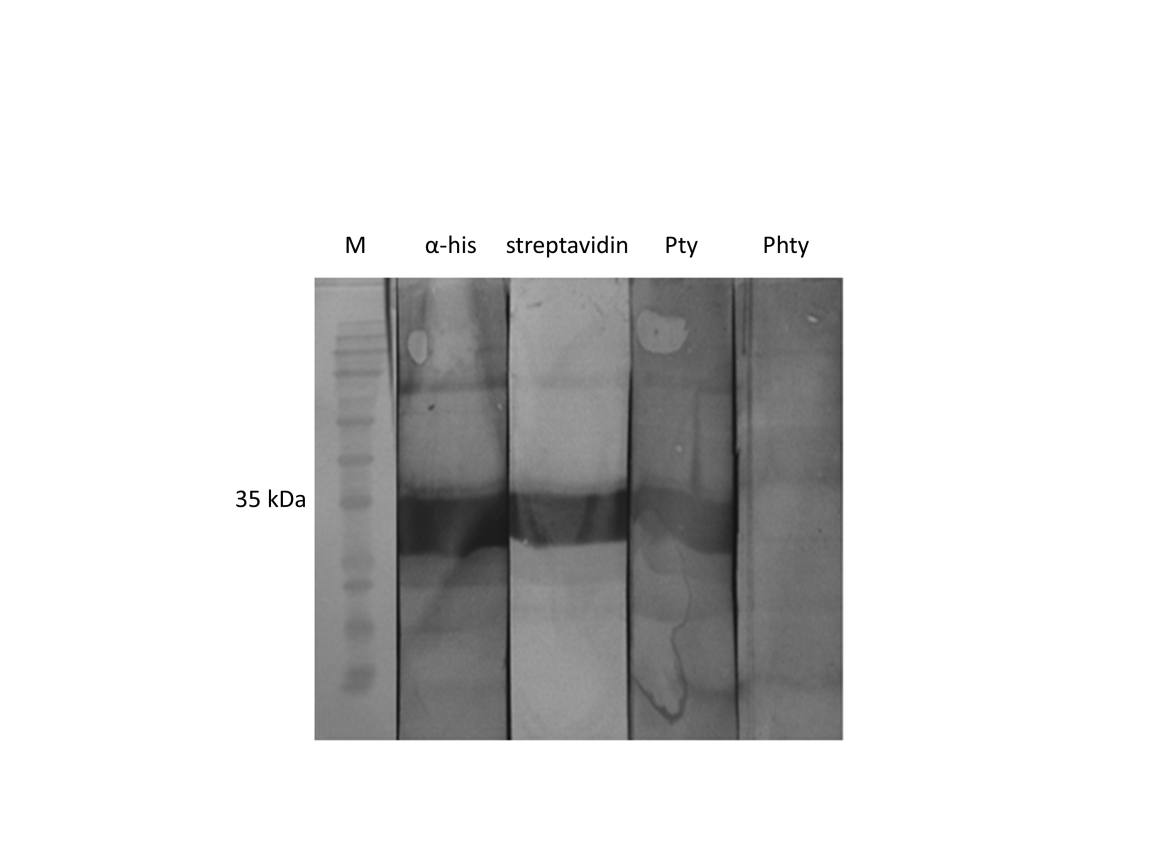


**Figure S2. Immunoblot assay of rHlyE.** The immunoblot shown with M indicates protein marker; α-His, rHlyE detected with α-His antibodies; Streptavidin, rHlyE detected with streptavidin-HRP; Pty, rHlyE detected by specific IgG in Pty sera; Phty, rHlyE detected with IgG in Phty sera.

**
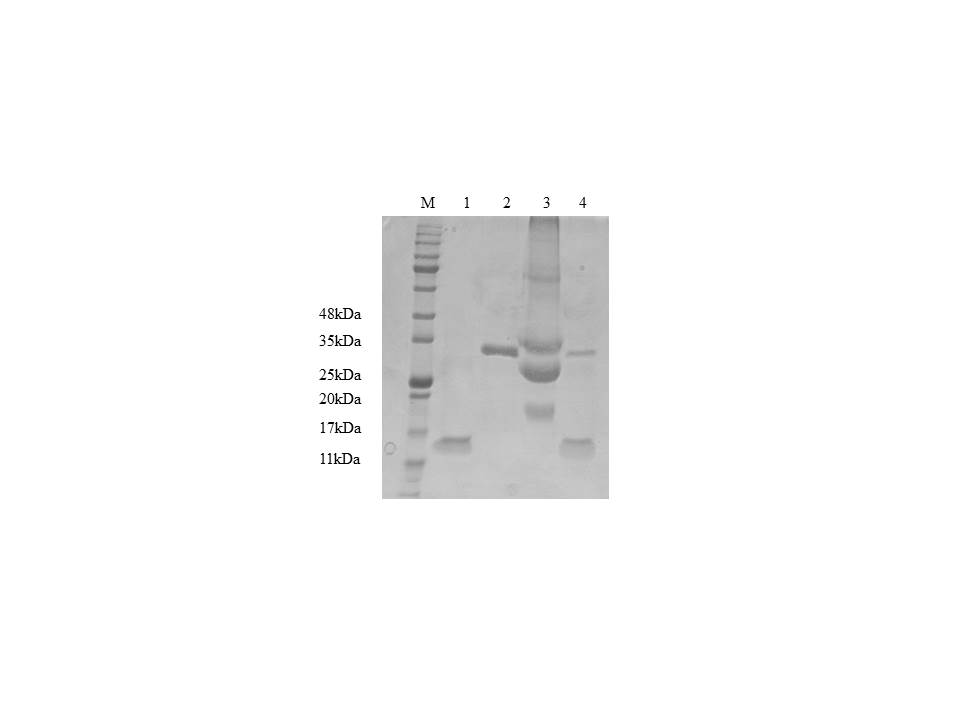
**

**Figure S3. Coomassie blue staining of a 12% SDS-polyacrylamide gel showing HlyE antigen coupled with streptavidin bead**. M indicates protein marker; lane 1, blank streptavidin bead; lane 2, purified protein of rHlyE; lane 3, blocking buffer (PTM); lane 4, streptavidin bead coupled with rHlyE.


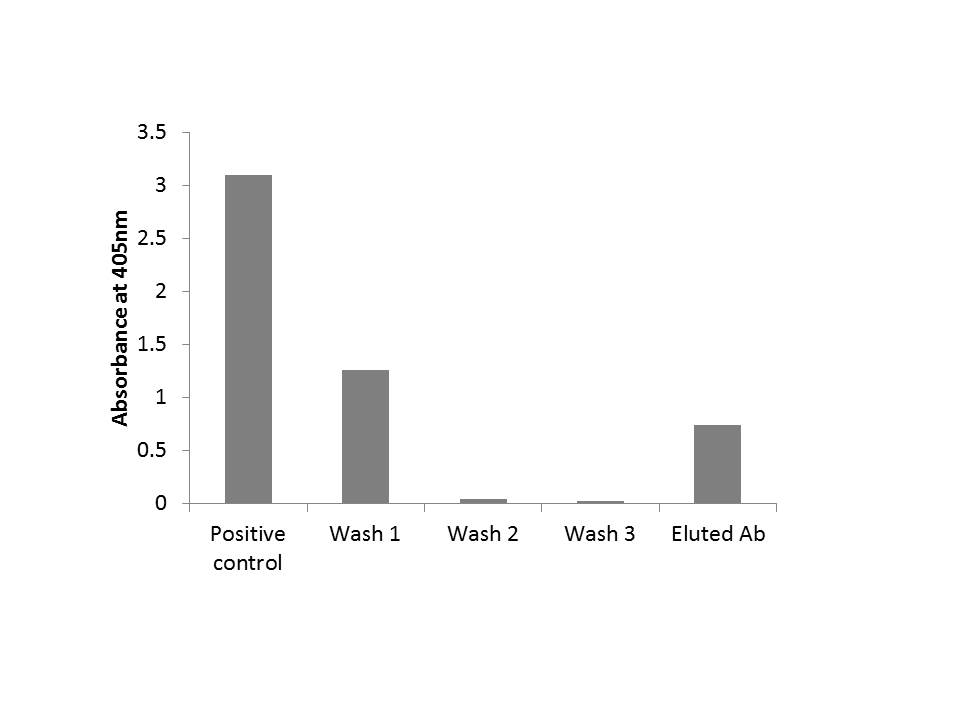


**Figure S4. ELISA result demonstrates the purified PcAb are specific to rHlyE antigen.** The positive control indicates binding between rHlyE protein with Pty sera.

10 20 30 40 50 60

Sequence MTGIFAEQTVEVVKSAIETADGALDLYNKYLDQVIPWKTFDETIKELSRFKQEYSQEASV

PepSurf -----------------------------------------------------EEEE-EE

EpiSearch -----------------------------------------E-EEE-EEEEEEEEEE-EE

CBTOPE EEEEEEEEEEEEEEEEEEEE--EE-----E-EEEEE-----------------EEEEEEE

Conf epitope -----------------------------------------------------EEEE-EE

70 80 90 100 110 120

Sequence LVGDIKVLLMDSQDKYFEATQTVYEWCGVVTQLLSAYILLFDEYNEKKASAQKDILIRIL

PepSurf E-----------------------------------------------------------

EpiSearch E-EE-EEE-E--------------------------------------------------

CBTOPE E--------------EEEEEEEEEEE--E-------------------------------Conf epitope E-----------------------------------------------------------

130 140 150 160 170 180

Sequence DDGVKKLNEAQKSLLTSSQSFNNASGKLLALDSQLTNDFSEKSSYFQSQVDRIRKEAYAG

PepSurf ------------------------------------------------------------

EpiSearch ----------------------E-EEE-EE-EEE-EE-EEEEEEE-EEEEEE---E----

CBTOPE -------EEEEEE---EEE-----E----E-EEEE-----------------E-E-----

Conf epitope ------------------------E----E-EEE--------------------------

190 200 210 220 230 240

Sequence AAAGIVAGPFGLIISYSIAAGVIEGKLIPELNNRLKTVQNFFTSLSATVKQANKDIDAAK

PepSurf ------------------------------------------------------------

EpiSearch ------EEEEE-E--------------E-E-EEE-EEEEEE-EE-E--------------

CBTOPE E-E----------------E-----------E------E---------EE-----EE---

Conf epitope -------------------------------E------E---------------------

250 260 270 280 290 300

Sequence LKLATEIAAIGEIKTETETTRFYVDYDDLMLSLLKGAAKKMINTCNEYQQRHGKKTLFEV

PepSurf ------------------------------------------------------------

EpiSearch ------------------------------------------------------------

CBTOPE -------E---------------------E---E-----------EE---EEE----E--

Conf epitope ------------------------------------------------------------

Sequence PDV

PepSurf ---

EpiSearch ---

CBTOPE ---

Conf epitope ---

**Figure S5. Predicted conformational epitope of HlyE.** The prediction is based on overlapped residues from EpiSearch and CBTOPE bioinformatics tools. PepSurf algorithm also fits certain residues of the conformational epitope predicted with blue colour indicates the potential conformational epitope residues

10 20 30 40 50 60

Sequence MTGIFAEQTVEVVKSAIETADGALDLYNKYLDQVIPWKTFDETIKELSRFKQEYSQEASV

BCPred ------------------------------------------------------------

ElliPro(L) ------------------------------------------------------------

L epitope ------------------------------------------------------------

70 80 90 100 110 120

Sequence LVGDIKVLLMDSQDKYFEATQTVYEWCGVVTQLLSAYILLFDEYNEKKASAQKDILIRIL

BCPred ------------------------------------------------------------

ElliPro(L) ------------------------------------------------------------

L epitope ------------------------------------------------------------

130 140 150 160 170 180

Sequence DDGVKKLNEAQKSLLTSSQSFNNASGKLLALDSQLTNDFSEKSSYFQSQVDRIRKEAYAG

BCPred ---------------------------------------------------EEEEEEEEE

ElliPro(L) -------------------------EEEEEEEEEEEEEEEEEEEEEEEEEEEEEEEEEEE

L epitope ---------------------------------------------------EEEEEEEEE

190 200 210 220 230 240

Sequence AAAGIVAGPFGLIISYSIAAGVIEGKLIPELNNRLKTVQNFFTSLSATVKQANKDIDAAK

BCPred EEEEEEEEEEE-------------------------------------------------

ElliPro(L) EEEEEEEE--EEEEEEEEEEEEEEEEEEEEEEE---------------------------

L epitope EEEEEEEE--E-------------------------------------------------

250 260 270 280 290 300

Sequence LKLATEIAAIGEIKTETETTRFYVDYDDLMLSLLKGAAKKMINTCNEYQQRHGKKTLFEV

BCPred ---------EEEEEEEEEEEEEEEEEEEE-----EEEEEEEEEEEEEEEEEEEE------

ElliPro(L) ---------------------------------------------------EEEEEEEEE

L epitope ---------------------------------------------------EEE------

Sequence PDV

BCPred ---

ElliPro(L) EEE

L epitope ---

**Figure S6. Predicted linear epitope of HlyE**. The prediction is based on overlapped residues from BcPreds algoritm and ElliPro(L) web tools. Blue colour residues are the synthesized linear epitope.

10 20 30 40 50 60

S Typhi ---------------MTGIFAEQTVEVVKSAIETADGALDLYNKYLDQVIPWKTFDETIK

S Paratyphi A ------------------------------------------------------------

E coli ------------------------------------------------------------

70 80 90 100 110 120

S Typhi ELSRFKQEYSQEASVLVGDIKVLLMDSQDKYFEATQTVYEWCGVVTQLLSAYILLFDEYN

S Paratyphi A --------YSQE-SVL--------------------------------------------

E coli --------YSQA-SVL--------------------------------------------

*

130 140 150 160 170 180

S Typhi EKKASAQKDILIRILDDGVKKLNEAQKSLLTSSQSFNNASGKLLALDSQLTNDFSEKSSY

S Paratyphi A ---------------------------------------S----A-DSQ-----------

E coli ---------------------------------------S----A-DSQ-----------

190 200 210 220 230 240

S Typhi FQSQVDRIRKEAYAGAAAGIVAGPFGLIISYSIAAGVIEGKLIPELNNRLKTVQNFFTSL

S Paratyphi A --------------GAAAGIVAG-----------------------N------Q------

E coli --------------GAAAGVVVG-----------------------K------Q------

* * *

250 260 270 280 290 300

S Typhi SATVKQANKDIDAAKLKLATEIAAIGEIKTETETTRFYVDYDDLMLSLLKGAAKKMINTC

S Paratyphi A ------------------------------------------------------------

E coli ------------------------------------------------------------

310

S_Typhi NEYQQRHGKKTLFEVPDV

S Paratyphi A ------------------

E coli ------------------

**Figure S7. Alignment of the predicted HlyE epitopes among different species.** Alignment of linear (blue) and conformational (green) epitope regions between *S.* Typhi, *S* Paratyphi A and *E coli* with difference in amino acids highlighted in yellow*.*


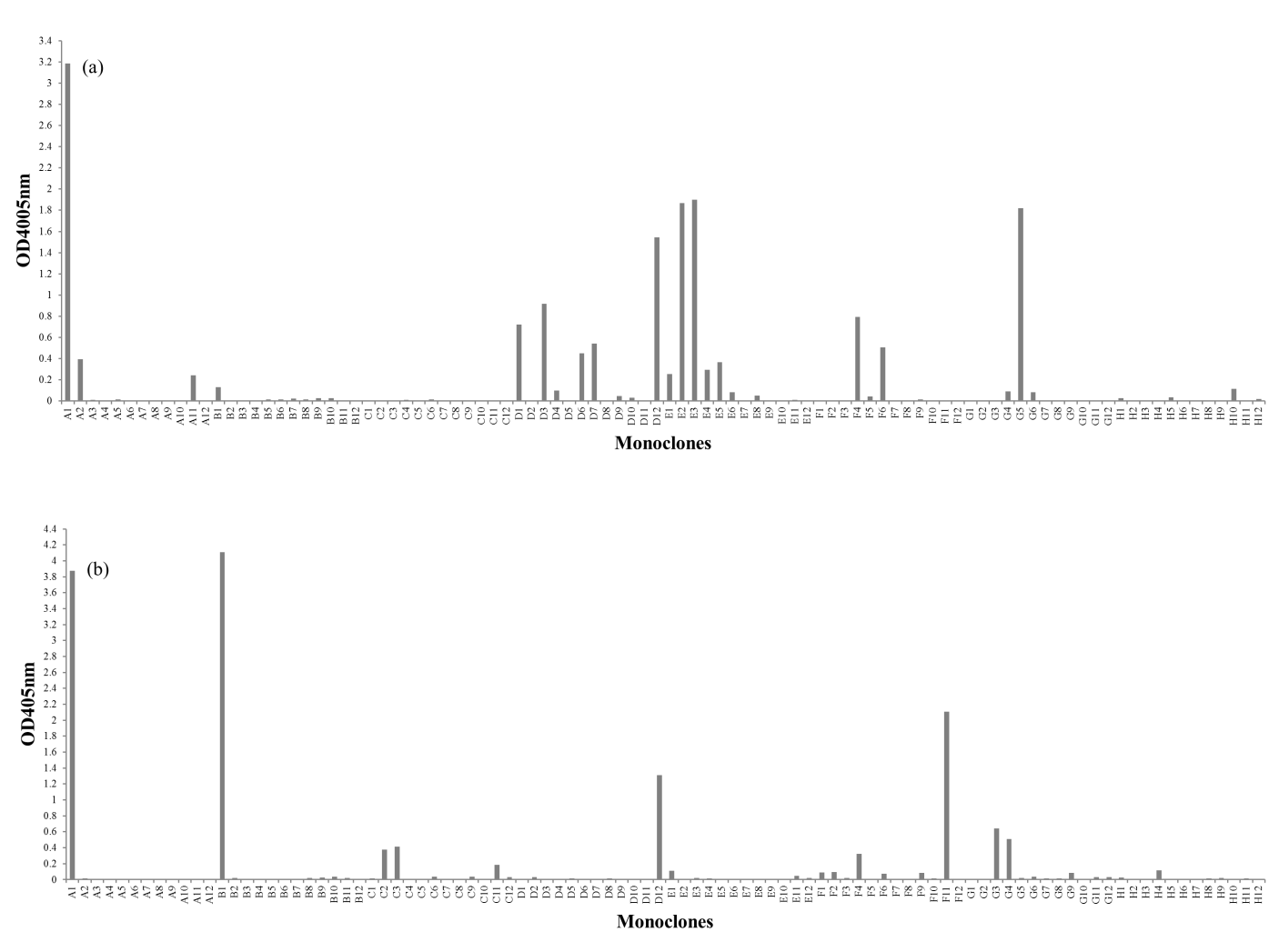


**Figure S8. Monoclonal ELISA result of mAbs against respective HlyE epitopes**. Naive antibody phage raised against (a) linear epitope and (b) conformational epitope with both A1 as positive control binding of anti-eGFP scFv monoclonal phage against recombinant eGFP, A3 as negative control binding against blank M13K07 phage.


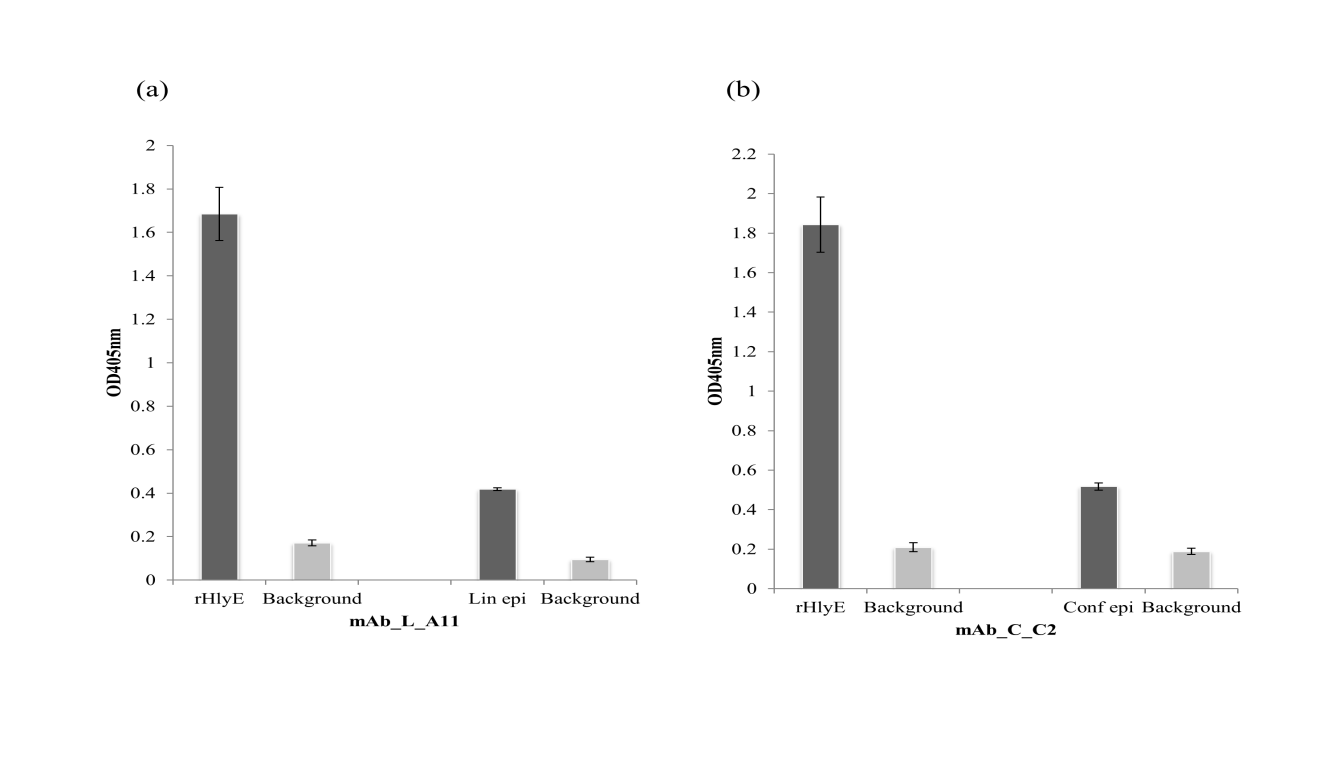


**Figure. S9** **Phage ELISA of mAbs against respective epitopes.** (a) mAb_L_A11 and (b) mAb_C_C2 scFvs against rHlyE and respective target epitope peptides with standard deviation shown in triplicate.


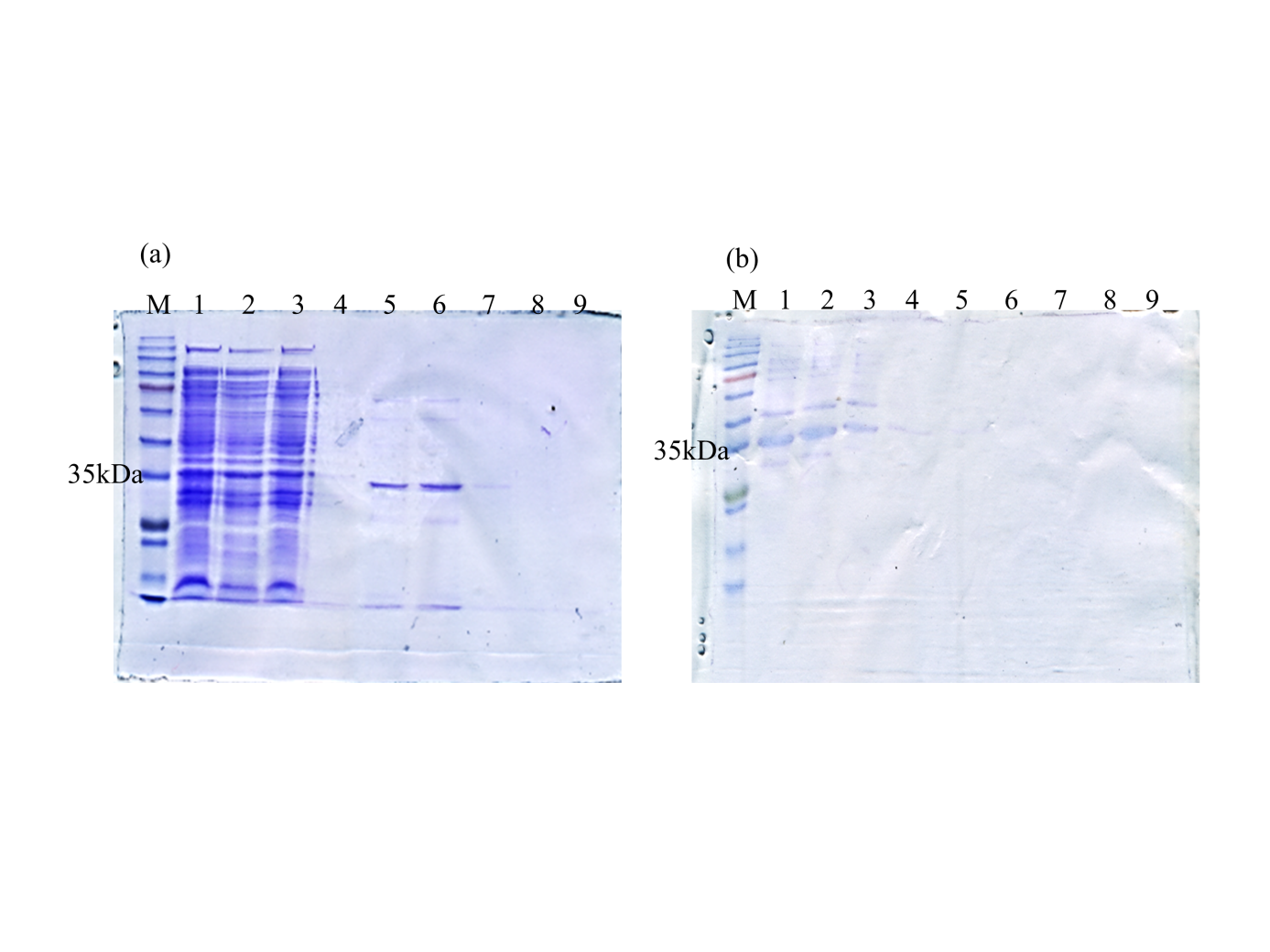


**Figure S10.** **Coomassie blue staining isolated monoclonal scFvs.** The 12% SDS-polyacrylamide gel showing (a) mAb_L_A11 and (b) mAb_C_C2 scFvs expression and purification with M indicates BLUelf prestained protein marker; lane 1, crude of lysate protein; lane 2, flow-through of the lysate protein; lane 3, first wash; lane 4, last wash; lane 5-9, lysates after passage through affinity column.


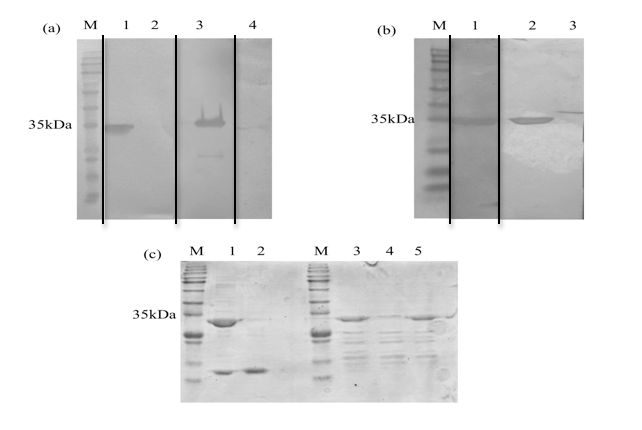


**Figure S11. Validation of isolated mAb_L_All and mAb_C_C2 scFvs.** (a) Denaturing immunoblot of mAb_L_A11 with M indicates protein marker; lane1, rHlyE detected with streptavidin-HRP; lane 2, rHlyE detected with anti-c-Myc Ab HRP, lane 3, target scFv detected with anti-c-myc Ab HRP; lane 4, rHlyE binding with target scFv and detected using anti-c-Myc Ab HRP; (b) denaturing immunoblot of mAb_C_C2 and with M indicates protein marker; lane1, rHlyE detected with streptavidin-HRP; lane 2, rHlyE detected with anti-His Ab, lane 3, mAb_C_C2 detected with anti-His Ab; (c) pull down assay of mAb_C_C2 with M indicates protein marker; lane 1 streptavidin bead coupled with rHlyE; lane 2, streptavidin bead with PTM; lane 3, eluted antibodies from rHllyE bead; lane 4, eluted antibodies from PTM bead; lane 5, eluted antibodies from conformational epitope coupled bead. Black lines indicate blots that were incubated and developed separately with different secondary antibodies.


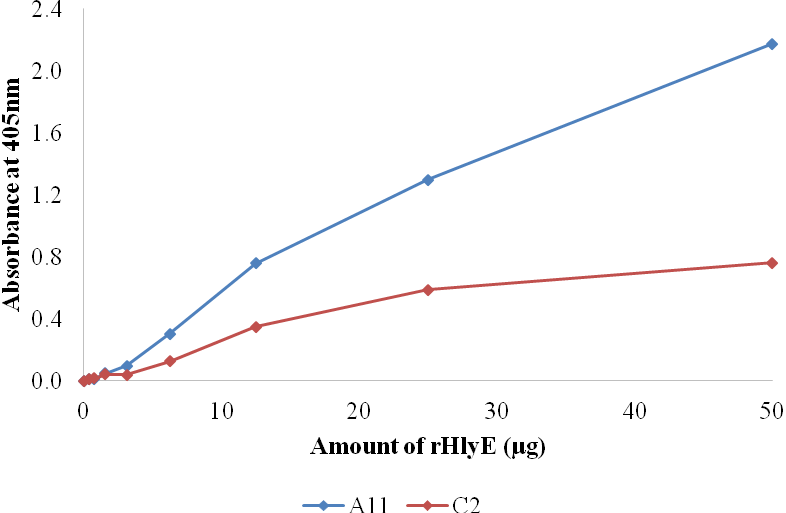


**Figure S12. ELISA result demonstrates the binding efficacy of mAb_L_A11 and mAb_C_C2 scFvs against rHlyE.**

Table S1 Result of phage enrichment titer for biopanning against anti-HlyE PcAb.

|  | **Input (CFU)** | **Output(CFU)** | **Ratio (Ouput/Input)** |
| --- | --- | --- | --- |
| **Round 1** | 2.0x10^12^ | 4.2x10^5^ | 2.1x10^-7^ |
| **Round 2** | 4.0x10^13^ | 4.0x10^7^ | 1.0x10^-6^ |
| **Round 3** | 4.0x10^13^ | 6.0x10^7^ | 1.5x10^-6^ |

Table S2 Enrichment of phage from each round of biopanning against HlyE linear and conformational epitope.

| **Linear epitope** | | | | **Conformational epitope** | | |
| --- | --- | --- | --- | --- | --- | --- |
|  | **Input (CFU)** | **Output (CFU)** | **Ratio (Ouput/Input)** | **Input (CFU)** | **Output**  **(CFU)** | **Ratio (Ouput/Input)** |
| **Round 1** | 1.1x10^10^ | 2.0x10^2^ | 1.8x10^-8^ | 1.3x10^10^ | 1.1x10^4^ | 8.4x10^-7^ |
| **Round 2** | 1.0x10^10^ | 1.0x10^4^ | 1.0x10^-6^ | 7.0x10^10^ | 2.0x10^6^ | 2.9x10^-5^ |
| **Round 3** | 8.0x10^9^ | 1.0x10^4^ | 1.3x10^-6^ | 1.3x10^10^ | 1.0x10^6^ | 7.7x10^-5^ |

Table S3 Unique CDRs region of mAbs isolated from naïve scFv library against respective rHlyE linear and conformational epitopes

| Mabs | Heavy Chain | | | Light Chain | | |
| --- | --- | --- | --- | --- | --- | --- |
|  | **CDR1** | **CDR2** | **CDR3** | **CDR1** | **CDR2** | **CDR3** |
| mAb_L_A11 | GYTFTSYG | ISAYNGNT | CARGGYCSSTSCYGFRFLEWGNWFDP | GSNIGSNP | RNT | AAWDDSLSGLV |
| mAb_C_C2 | GYTFTGYY | IIPIFGTA | ARCDYDILTGLYYYYGMDV | QSLLHSNGYNY | LGS | MQALQAPRT |

**Table S4: Mutational analysis of conformational epitope by alanine substitution.**

| **Index** | **Wild residue** | **Residue position** | **Mutant residue** | **Predicted ΔΔG** | **Outcome** |
| --- | --- | --- | --- | --- | --- |
| 1 | Y | 54 | A | -0.331 | Reduced affinity |
| 2 | S | 55 | A | 0.501 | Increased affinity |
| 3 | Q | 56 | A | 0.482 | Increased affinity |
| 4 | E | 57 | A | -0.569 | Reduced affinity |
| 5 | S | 59 | A | 0.662 | Increased affinity |
| 6 | V | 60 | A | 0.54 | Increased affinity |
| 7 | L | 61 | A | 0.563 | Increased affinity |
| 8 | S | 145 | A | -0.068 | Reduced affinity |
| 9 | D | 152 | A | -0.47 | Reduced affinity |
| 10 | S | 153 | A | 0.031 | Increased affinity |
